# Advancements in artificial intelligence for cancer diagnosis and prognosis prediction: current applications and emerging opportunities

**DOI:** 10.3389/fcell.2026.1769097

**Published:** 2026-04-22

**Authors:** Qingmiao Shi, Na Lou, Chen Xue

**Affiliations:** 1 Department of Infectious Diseases, The First Affiliated Hospital, College of Clinical Medicine, Henan University of Science and Technology, Luoyang, China; 2 Department of Infectious Diseases, The First Affiliated Hospital of Zhengzhou University, Zhengzhou, China; 3 State Key Laboratory for Diagnosis and Treatment of Infectious Diseases, National Clinical Research Center for Infectious Diseases, National Medical Center for Infectious Diseases, Collaborative Innovation Center for Diagnosis and Treatment of Infectious Diseases, The First Affiliated Hospital, Zhejiang University School of Medicine, Hangzhou, China

**Keywords:** artificial intelligence, cancer screening, challenges and opportunities, diagnosis accuracy, prognosis prediction

## Abstract

Cancer continues to be a leading cause of mortality worldwide, presenting substantial challenges to public health systems. The traditional approaches to cancer diagnosis and prognosis prediction exhibit certain limitations with respect to accuracy, comprehensiveness, dynamic monitoring, and personalization. With the advancement of artificial intelligence (AI) technologies, novel diagnostic and predictive methods are increasingly addressing these shortcomings. This review provides a comprehensive overview of the primary AI algorithms applied in oncology, including machine learning, deep learning, and large language models. It further examines the distinctive characteristics and appropriate use cases of AI algorithms, highlighting their specific roles in cancer screening, diagnostic accuracy, and outcome forecasting. Additionally, the review discusses emerging trends and persistent challenges, aiming to provide actionable insights that support clinical decision-making and advance scientific innovation in this rapidly evolving field. In conclusion, this review systematically outlines recent advances in AI applications for cancer diagnosis and prognostic prediction, with the objective of facilitating a transformative shift in oncology from experience-based practices toward data-driven precision medicine.

## 1 Introduction

Cancer, one of the leading causes of death worldwide, is projected to account for over 35 million new cases by 2050, posing a significant challenge to global public health systems (Bray et al., 2024). In conventional cancer diagnosis and treatment, clinicians conduct comprehensive analyses of heterogeneous data sources, including computed tomography (CT) and magnetic resonance imaging (MRI) imaging, histopathological slides, laboratory test results, and electronic medical records (Hajjawi et al., 2024). However, this process is not only time-consuming and labor-intensive but also limited in its capacity to identify complex patterns within high-dimensional datasets, while remaining vulnerable to subjective interpretation.

In recent years, the integration of artificial intelligence (AI) into healthcare has opened new avenues for addressing these limitations (Zhang C. et al., 2023). AI, as an emerging scientific discipline, focuses on the investigation and development of theories, methods, technologies, and application systems designed to simulate, extend, and enhance human cognitive capabilities (Hamet and Tremblay, 2017). By integrating multimodal data, including medical images, pathological slides, multi-omics profiles, and clinical records, AI has catalyzed a paradigm shift in oncology from experience-based decision-making to data-driven precision medicine (Kumar et al., 2022; Tiwari et al., 2025). Notably, in the domains of cancer diagnosis and prognostic prediction, AI has demonstrated considerable potential, significantly improving diagnostic accuracy, therapeutic planning, and outcome forecasting.

This review systematically outlines recent advancements in AI applications for cancer diagnosis and prognostic prediction, examines the characteristics and appropriate use cases of various AI algorithms, and highlights their specific roles in cancer screening, diagnostic refinement, and prognostic prediction. It further discusses emerging trends and persistent challenges in the field. By outlining the technological pathways and application prospects in this rapidly evolving field, this review aims to provide actionable insights to support clinical practice and advance scientific innovation in oncology.

## 2 Types of AI algorithm applied in oncology

**FIGURE 1:**
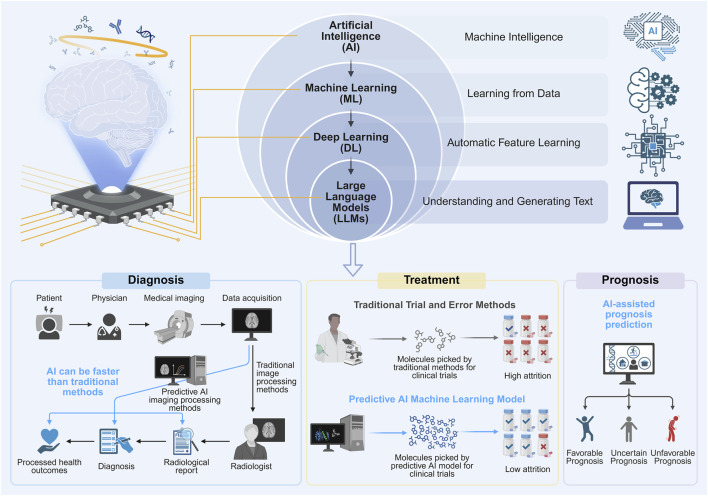
Types of AI and its applications in cancer diagnosis, treatment and prognosis. This figure illustrates the technical evolution of Artificial Intelligence (AI) from Machine Learning (ML) and Deep Learning (DL) to Large Language Models (LLMs). It highlights the core advantages of AI in accelerating diagnostic speed, enhancing therapeutic efficiency, and accurately predicting patient outcomes.

The evolution of modern AI has progressed through several pivotal stages, from traditional machine learning (ML) to deep learning (DL), and more recently to large language models (LLMs). These technologies are being integrated into various domains of oncology at an accelerating pace, demonstrating transformative potential across basic research, clinical diagnosis, and therapeutic decision-making. Owing to their distinct learning mechanisms and data processing capabilities, each class of algorithm plays a critical role in different phases of cancer care. This section provides an overview of the key characteristics and application contexts of ML, DL, and LLMs in oncology (Figure 1).

### 2.1 ML

As a data-driven methodology, ML enables the identification of complex patterns and the generation of predictive insights from large-scale clinical datasets, thereby supporting clinical decision-making (Harkos et al., 2025). Its applications in oncology are broad, spanning the analysis of medical imaging, molecular profiles, and electronic health records (EHRs) for tasks including cancer detection, diagnosis, prognosis prediction, and assessment of treatment response (Gao et al., 2023; Swanson et al., 2023). Furthermore, ML holds significant promise in drug discovery and development. For example, ML optimizes nanoparticle design to improve targeted drug delivery efficiency (Wang Q. et al., 2025).

### 2.2 DL

As an advanced subset of ML, DL is distinguished by its capacity for automatic feature extraction, making it particularly effective in processing high-dimensional and unstructured data such as medical images and textual reports (Chen Z. H. et al., 2021; Shrestha, 2025). In radiology, DL models have achieved performance on par with or exceeding that of human experts in detecting tumors in mammography and breast ultrasound (Dan et al., 2024; Xue et al., 2022). In neuro-oncology, DL facilitates brain tumor segmentation, classification, and outcome prediction, contributing to the advancement of precision medicine (Dorfner et al., 2025). Notably, DL models can infer key molecular characteristics, such as specific genetic mutations and tumor mutational burden, directly from routine hematoxylin and eosin (H&E)-stained histopathological slides, enabling a concept known as “digital biopsy” (Lee and Jang, 2022). More recently, foundational models such as CHIEF have emerged, demonstrating generalizable performance in cancer evaluation and prognostic prediction across diverse cancer types and healthcare institutions (Wang et al., 2024). Models such as UNI (Chen R. J. et al., 2024) and Phikon (Petzold et al., 2026; Şeker et al., 2025) learn generalizable representations from massive histopathological images through self-supervised learning, reducing the dependence on annotated data. The Virchow model (Khalili and Ciompi, 2024; Vorontsov et al., 2024) has demonstrated remarkable pan-cancer detection capabilities, particularly for rare malignancies. Furthermore, cutting-edge multimodal foundation models like PRISM (Shi et al., 2025) are pioneering the seamless integration of histopathological images with complex genomic profiles. In 2025, Deepath-MSI (Feng et al., 2025) was approved by China’s National Medical Products Administration (NMPA) as a Class III innovative medical device for microsatellite instability detection in colorectal cancer (CRC) (AUROC 0.98), marking a critical transition from laboratory algorithms to clinically approved, regulatory-recognized applications.

### 2.3 LLMs

The emergence of LLMs represents a paradigm shift in AI’s ability to interpret and generate human language, particularly in handling unstructured clinical text. Moreover, LLMs are increasingly being combined with vision-based networks to form multimodal foundation models (Truhn et al., 2024). With the capability to understand and produce natural language, LLMs are expanding rapidly into oncology applications. They can parse EHRs to provide decision support for prognosis estimation, treatment recommendation, and clinical trial matching (Verlingue et al., 2024). They can also efficiently extract structured information, such as TNM staging, from pathology reports and other texts (Wiest et al., 2025). By leveraging vast repositories of biomedical literature and real-world patient data, LLMs can develop risk stratification tools that outperform conventional clinical scoring systems (Jairath et al., 2025). Additionally, they can simplify technical terminology in radiology reports and provide multilingual translations to enhance patient understanding and accessibility (Tordjman et al., 2025). The integration of LLMs with visual models into unified multimodal architectures signals a future in which AI systems can simultaneously interpret heterogeneous data streams, including text and imaging, enabling more comprehensive and personalized clinical decision support (Luo et al., 2025).

In summary, ML, DL, and LLMs are transforming oncology research and clinical practice in complementary ways. In this domain, ML serves as the foundational tool for data analysis and prediction, DL represents a more advanced technology capable of automatic learning from complex data, and LLMs are the latest tools specifically designed for processing and utilizing massive textual information, now being integrated into multimodal clinical decision support systems. Their synergistic development and convergence are collectively driving innovation in precision oncology, paving the way for more accurate, efficient, and individualized cancer care.

## 3 AI applications in cancer screening

**FIGURE 2:**
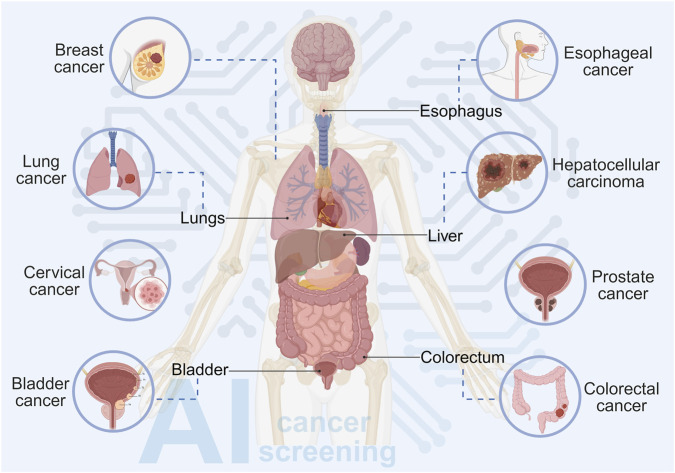
Cancer types screened through AI-based approaches. Currently, AI is widely utilized for the early identification of multiple prevalent malignancies, including breast, lung, esophageal, hepatocellular, prostate, colorectal, bladder, and cervical cancers, demonstrating its versatility in systemic cancer screening.

In recent years, AI technologies, particularly ML and DL, have achieved substantial advancements in early cancer screening, demonstrating transformative potential for conventional medical paradigms. By enabling automated and rapid analysis of complex biomedical data, AI offers innovative solutions to enhance the efficiency, accuracy, and accessibility of cancer screening programs. The following sections summarize the specific applications of AI across various cancer types (Figure 2).

### 3.1 CRC screening

Emerging evidence indicates that AI technologies exhibit significant value in the screening of CRC (Goyal et al., 2020; Han et al., 2025; Momen-Roknabadi et al., 2025). In histopathological assessment, Ho et al. developed a composite AI model integrating a DL-based Faster R-CNN with a classical ML classifier to identify malignancies in whole slide images (WSIs) of colorectal biopsies. This framework demonstrated exceptionally high sensitivity for detecting high-grade lesions, positioning it as a robust decision-support tool for pathologists (Ho et al., 2022). Furthermore, Pan et al. employed a “Bionic Glycome” quantitative approach combined with ML to construct a diagnostic model based on serum N-glycan profiles, capable of differentiating between healthy individuals, those with advanced adenomas, and patients with CRC. This approach presents a promising avenue for non-invasive population-level screening (Pan et al., 2021).

### 3.2 Esophageal cancer screening

AI research in esophageal cancer screening has primarily focused on esophageal squamous cell carcinoma (ESCC) (Li S. W. et al., 2024; Meng et al., 2022; Yan S. Y. et al., 2025). Yan et al. detailed the application of ML, DL, and transfer learning in early ESCC detection, highlighting AI’s potential to optimize screening protocols, improve lesion identification, and enable risk prediction through the integration of genomic, imaging, and clinical data (Yan S. Y. et al., 2025). Additionally, for Barrett’s esophagus, a precancerous condition, Bouzid et al. developed a weakly supervised DL model that enables effective screening using standard H&E-stained histopathological slides. This model has the potential to reduce pathologists’ workload by nearly 50%, thereby facilitating large-scale population-based screening initiatives (Bouzid et al., 2024).

### 3.3 Lung cancer screening

The application of AI in lung cancer screening is particularly extensive, spanning multiple stages of the screening workflow. Chamberlin et al. developed an AI-powered convolutional neural network (CNN) prototype capable of automatically detecting pulmonary nodules and quantifying coronary artery calcium volume on low-dose CT (LDCT) scans with high precision. Notably, these quantitative outputs have demonstrated prognostic significance for major cardiopulmonary adverse events (Chamberlin et al., 2021). Cai et al. introduced a multimodal early detection platform that integrates a multiplexed laser-induced graphene immunosensor with ML, combining proteomic data, DL-derived CT imaging features, and clinical variables to significantly enhance diagnostic performance for early-stage lung cancer (Cai et al., 2025). Importantly, Chao et al. demonstrated that DL models can leverage LDCT data from lung cancer screening to simultaneously predict cardiovascular disease risk, enabling a “one scan, dual screening” strategy (Chao et al., 2021). Moreover, researchers have developed a 3D plasmonic hexaplex nanostructure sensor integrated with ML for label-free detection in saliva samples, offering a non-invasive method for early lung cancer screening (Linh et al., 2024).

### 3.4 Breast cancer screening

AI has become deeply embedded in breast cancer imaging analysis. Bahl et al. compared a conventional computer-aided detection system applied to synthetic 2D mammography with a DL-based AI algorithm applied to digital breast tomosynthesis (DBT) images, the latter exhibited superior performance in terms of AUC, sensitivity, and specificity (Bahl et al., 2025). Wang et al. enhanced the YOLACT architecture by incorporating a hard-sample mining mechanism, improving detection accuracy for both typical and challenging cases in breast MRI (Wang and Wang, 2023). Chen et al. investigated a multimodal DL model that integrates mammographic and ultrasonographic data, finding that its overall performance surpasses that of single-modality approaches, thus offering new strategies to improve screening accuracy (Chen J. et al., 2025).

### 3.5 Cervical cancer screening

Automated cytological image analysis powered by AI has emerged as a key focus in cervical cancer screening. Cheng et al. developed a progressive lesion cell recognition method and a recurrent neural network-based WSI classification model, which outperformed average cytopathologists in large-scale, multi-center validation studies (Cheng et al., 2021). Studies by Kanavati et al. (2022) and Wang C. W. et al. (2021) further confirmed the high diagnostic accuracy of DL models in distinguishing neoplastic from non-neoplastic lesions in cervical liquid-based cytology (LBC) WSIs. Fu et al. proposed a cross-modal integration framework that combines a DL model analyzing colposcopic images with cytology and HPV testing results, achieving state-of-the-art performance in cervical cancer screening (Fu et al., 2022). Additionally, Arrivillaga et al., guided by clinical needs and human factors engineering principles, developed CITOBOT, a portable AI-enabled cervical cancer screening device designed for deployment in resource-limited settings (Arrivillaga et al., 2024).

### 3.6 Urinary system cancers screening

AI technologies are providing powerful tools for non-invasive or minimally invasive screening of urinary system cancers. For urothelial carcinoma, Xin et al. combined digital holographic flow cytometry with ML and DL algorithms to achieve high-accuracy identification of malignant cells in urine samples (Xin et al., 2024). Tsuneki et al. trained a DL model to classify urine LBC WSIs, accurately differentiating neoplastic from non-neoplastic cases (Tsuneki et al., 2022). In bladder cancer screening, Su et al. integrated digital microfluidics, fluorescence lifetime imaging microscopy, and DL to develop a highly accurate, non-invasive diagnostic approach (Su et al., 2024). For prostate cancer, Kim et al. developed a non-invasive method combining multiplex urinary biomarker sensing with AI-driven analysis, achieving over 99% accuracy in patient classification (Kim et al., 2021).

### 3.7 Other cancers screening

AI also demonstrates diverse utility in the screening of other malignancies. Fanet al. achieved hepatocellular carcinoma (HCC) screening by analyzing urine samples using atmospheric pressure glow discharge mass spectrometry coupled with ML (Fan et al., 2023). In hematologic oncology, Qin et al. applied ML to screen biomarkers across 13 programmed cell death (PCD) pathways, constructing a gene signature with utility for prognosis prediction and therapeutic guidance in acute myeloid leukemia (AML) (Qin et al., 2024). In drug discovery, Valentini et al. identified novel small molecules targeting the anti-apoptotic Bcl-2 protein family through ML-based virtual screening, subsequently validating their anti-tumor efficacy in preclinical models (Valentini et al., 2022).

In summary, AI technologies have been extensively integrated into the screening processes for a wide range of cancers, with applications spanning medical image interpretation, liquid biopsy, histopathological evaluation, and biomarker discovery. These advances collectively underscore the critical role of AI in enhancing screening efficiency, diagnostic accuracy, and the feasibility of early cancer detection.

## 4 AI applications in cancer diagnosis

**FIGURE 3:**
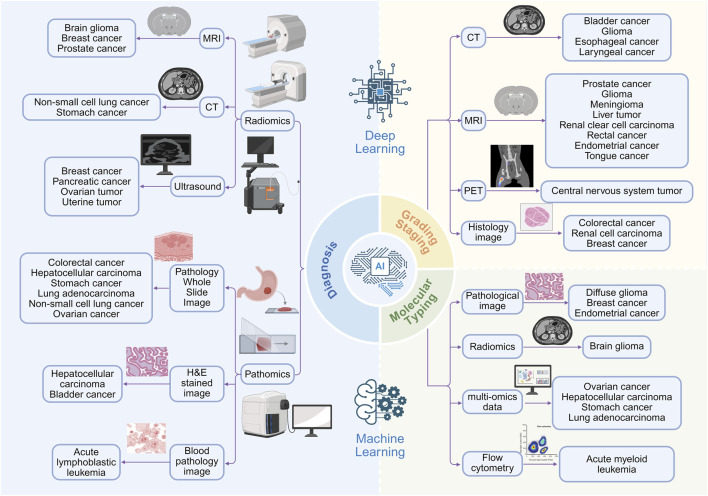
Applications of AI in cancer diagnosis, grading and staging, molecular typing. By integrating multi-source data, such as MRI, CT, PET, ultrasound, and histopathological images, AI facilitates not only tumor diagnosis but also precise pathological grading and clinical staging. Furthermore, combined with multi-omics data, it enables accurate molecular typing to support personalized treatment decision-making.

AI, particularly DL, is fundamentally characterized by the construction of multi-layer neural networks that autonomously learn hierarchical feature representations from raw data (Roy et al., 2020). The systematic review by Kumar et al. affirmed the high performance of AI in cancer diagnosis, yet pointedly noted that technological advances have not translated into a reduction in cancer mortality, highlighting the persistent challenge of insufficient clinical translation (Kumar et al., 2022). Lipkova et al., in turn, charted a path forward, emphasizing that multimodal data integration, combining diverse data sources such as imaging, pathology, and genomics, can enhance model robustness and interpretability, bringing AI closer to real-world clinical practice and offering the potential to uncover novel biomarkers (Lipkova et al., 2022). The following sections will elaborate on the specific applications of AI in the diagnosis of various cancers (Figure 3; Table 1).

**TABLE 1:** The application of AI in cancer diagnosis, staging and grading, and molecular classification.

Category	Data source		Cancer type	AI method	Function	References
Diagnosis	Radiomics	Multimodal MRI, liquid biopsy	Glioma	Deep learning	Achieves preoperative non-invasive “integrated diagnosis”	Hu et al. (2023)
		Low-Dose CT	Non-small cell lung cancer (NSCLC)	Deep learning	Effectively predicts distal metastasis in NSCLC	Song et al. (2024)
		Ultrasound	Breast cancer	Deep learning	Demonstrates high accuracy and strong generalizability on breast ultrasound images	Wang et al. (2025c)
		Multiparametric MRI	Breast cancer	Machine learning	The multiparametric MRI radiomics model shows superior performance in differentiating benign from malignant breast lesions	Daimiel Naranjo et al. (2021)
		B-mode ultrasound, Nakagami images	Breast cancer	Multimodal Deep learning	Enhances diagnostic efficacy	Muhtadi and Gallippi (2025)
		Digital breast tomosynthesis (DBT)	Breast cancer	Machine learning	Accurately diagnoses architectural distortion on DBT images, potentially avoiding unnecessary biopsies	Chen et al. (2022)
		Contrast-enhanced ultrasound	Pancreatic cancer	Deep learning	Effectively assists in differentiating pancreatic ductal adenocarcinoma from chronic pancreatitis	Tong et al. (2022)
		Transvaginal ultrasound	Ovarian tumor	Deep learning	Diagnostic performance surpasses individual models and even expert evaluation	Zhou et al. (2025)
		MRI	Prostate cancer	Deep learning	Improves the accuracy and robustness of prostate cancer diagnosis	Horasan and Güneş (2024)
		CT	Gastric cancer	Deep learning	Differentiates early-stage gastric cancer without lymph node metastasis	He et al. (2025)
		Ultrasound	Soft Tissue tumors	Deep learning	Accurately diagnoses benign and malignant soft tissue tumors	Dai et al. (2024)
		Ultrasound	Uterine tumors	Machine learning	Effectively differentiates uterine sarcomas from benign leiomyomas	Chiappa et al. (2021)
		CT	Malignant pleural effusion	Deep learning	Assists in diagnosing malignant pleural effusion	Ozcelik et al. (2023)
		Dual-modality ultrasound	Cervical lymphadenopathy	Deep learning	Accurately identifies etiology and narrows the diagnostic gap between physicians of different experience levels	Zhu et al. (2022)
​	Pathomics	Pathological whole-slide image	Colorectal cancer	Deep learning	Shows high concordance with pathologists, handles vast numbers of slides, and reduces diagnostic burden	Wang et al. (2021b)
		H&E-stained image	Hepatocellular carcinoma (HCC)	Deep learning	Enables efficient and accurate multi-class classification of HCC subtypes	Aatresh et al. (2021)
		Pathological whole-slide image	HCC	Deep learning	Enables precise diagnosis and quantitative assessment of microvascular invasion, improving diagnostic efficiency and consistency	Zhang et al. (2024a)
		Pathological whole-slide image	Gastric cancer	Deep learning	Non-cancerous peritumoral tissue possesses diagnostic value for gastric cancer	Li et al. (2025c)
		Pathological whole-slide image	Lung adenocarcinoma (LUAD)	Deep learning	Identifies tumor invasiveness in LUAD, assisting pathologists in improving diagnostic accuracy	Du et al. (2025)
		Pathological whole-slide image	NSCLC	Explainable Deep learning	Distinguishes NSCLC subtypes and provides visual reports for decision-making, enhancing clinical utility	Civit-Masot et al. (2022)
		Histopathological image	Acute lymphoblastic leukemia (ALL)	Deep learning and Machine learning	Enables automatic screening and classification of ALL histopathological images with high accuracy	Mehan (2025)
		H&E-stained image	Bladder cancer	Machine learning	Diagnoses bladder cancer with high accuracy; the generated risk score is an independent prognostic factor	Chen et al. (2021a)
		Pathological whole-slide image	Ovarian carcinoma	Deep learning	Improves the accuracy and consistency of pathological diagnosis	Farahani et al. (2022)
Staging and Grading	CT		Bladder cancer	Machine learning	Preoperative staging: predicting muscle invasion	Xiong et al. (2024)
			Bladder cancer	Machine learning	Preoperative staging: distinguishing T2 from T3 stage	Lisson et al. (2025)
			Upper tract urothelial carcinoma	Machine learning	Tumor grading	Al Mopti et al. (2025)
			Glioma	Machine learning	Tumor grading: differentiating low-grade vs. high-grade	Bilgin et al. (2025)
			Esophageal cancer	Deep learning and machine learning	Tumor grading	Alsallal et al. (2025)
​	​		Laryngeal carcinoma	Deep learning and machine learning	Preoperative staging: differentiating Stage I-II from III-IV	Chen et al. (2023)
			Thymic epithelial tumors	Machine learning	Predicting WHO stage and histological subtype	Zhang et al. (2025)
	MRI		Prostate cancer	Machine learning and deep learning	Predicting postoperative Gleason grading	Guo et al. (2025)
			Glioma	Machine learning	Tumor grading: differentiating low-grade vs. high-grade	Du et al. (2023)
			Glioma	Machine learning	Tumor grading: differentiating low-grade vs. high-grade	Hashido et al. (2021)
			Glioma	Machine learning	Tumor detection and grading	Chen et al. (2021b)
			Meningioma	Deep learning	Tumor grading: differentiating low-grade vs. high-grade	Zhang et al. (2024c)
			Meningioma	Deep learning and machine learning	Tumor grading: differentiating low-grade vs. high-grade	Yang et al. (2022)
			Liver tumors	Machine learning and Deep learning	LI-RADS grading	Du et al. (2022)
			Clear cell renal cell carcinoma	Machine learning	Predicting preoperative WHO/ISUP nuclear grading	Yang et al. (2024a)
			Rectal cancer	Machine learning	Predicting lymph node stage: N0/N1 vs. N2	Qubie et al. (2025)
			Endometrial cancer	Deep learning	Automatic early-stage classification: Stage IA vs. IB	Mao et al. (2022)
			Tongue cancer	Deep learning	Automatic T-staging	Lu et al. (2025)
	PET		Central nervous system tumors	Machine learning	Tumor grading: differentiating low-grade vs. high-grade	Russo et al. (2021)
	Histopathology		Colorectal cancer	Deep learning	Pathological staging: differentiating Stage I-II from Stage III	Tan et al. (2024)
			Renal cell carcinoma	Deep learning	Automatic tumor grading	Chanchal et al. (2023)
			Breast cancer	Deep learning	Tumor grading and prognosis prediction	Wetstein et al. (2022)
Molecular Classification	Pathology imaging		Diffuse glioma	Deep learning	Developed DeepGlioma system for rapid prediction of IDH, 1p/19q, and ATRX mutations	Hollon et al. (2023)
			Breast cancer	Deep learning	Molecular subtype classification using whole-slide images, with attention heatmaps revealing morphological features	Jang et al. (2025)
			Endometrial cancer	Deep learning	Prediction of POLEmut, MMRd, p53abn, and NSMP molecular subtypes based on H&E slides	Fremond et al. (2023)
			Spinal ependymoma	Deep learning	Prediction of DNA methylation-based molecular subtypes: SP-EPN and MPE from H&E slides	Schumann et al. (2024)
	Radiomics		Glioma	Machine learning	Applied point spread function deconvolution to optimize PET imaging features, improving molecular subtype performance	Ahrari et al. (2022)
			Glioma	Machine learning	Integrated RNAseq and radiomics features to predict IDH and ATRX mutations and 1p/19q co-deletion	Shboul et al. (2021)
	Multi-omics data		Ovarian cancer	Machine learning	Integrated multi-omics data to identify consensus molecular subtypes and construct prognostic and immunotherapy response prediction models	Ren et al. (2025)
			HCC	Machine learning	Performed molecular subtyping to distinguish tumor microenvironment characteristics, predicting prognosis and guiding treatment selection	Chen et al. (2024b)
			HCC	Machine learning	Subtyping based on multi-dimensional molecular feature scores to predict sensitivity to immunotherapy and targeted therapy	Li et al. (2024b)
			Gastric cancer	Machine learning	Identified three molecular subtypes associated with prognosis and chemotherapy sensitivity	Li et al. (2023)
			Esophagogastric adenocarcinoma	Machine learning	Validated the prognostic value of TCGA molecular subtypes and predicted treatment response	Walch et al. (2025)
			Lung adenocarcinoma	Machine learning	Established an epigenetic molecular classification system to predict prognosis and immunotherapy response	Wang et al. (2025a)
​	​		Soft tissue sarcoma	Machine learning	Conducted molecular subtyping based on m6A-related metabolic pathways and constructed an m6A metabolic score	Huang et al. (2022)
	Flow cytometry		Acute myeloid leukemia	Deep learning	Automated diagnosis and prediction of key molecular events including PML::RARA, RUNX1:RUNX1T1 fusions, and NPM1 mutations	Lewis et al. (2024)

### 4.1 Diagnosis

AI technologies, particularly ML, DL, and LLMs, are fundamentally transforming the landscape of cancer diagnosis. Their applications are primarily centered on two rapidly evolving domains: radiomics and pathomics, which enable the development of high-precision diagnostic models through the extraction of deep, subvisual features from medical imaging data that are imperceptible to the human eye.

#### 4.1.1 Radiomics

Radiomics entails the high-throughput extraction and analysis of quantitative features from medical images such as CT, MRI, and ultrasound. When integrated with AI algorithms, radiomic approaches support diagnostic decision-making, risk stratification, and outcome prediction, demonstrating broad clinical applicability.

In central nervous system (CNS) tumors, a recent study proposed a multi-task DL-based radiomic model designed to integrate multi-modal MRI with liquid biopsy data, aiming to achieve a preoperative, non-invasive “integrated diagnosis” of glioma aligned with the 2021 WHO Classification of Tumours of the CNS (Hu et al., 2023).

In the field of thoracic oncology, significant progress has been made in the diagnosis of pulmonary nodules, though methodological choices and validation rigor vary across studies. One study developed a computer-aided diagnosis framework that combines DL architectures with radiomic features to effectively differentiate benign from malignant ground-glass nodules, with the fused model outperforming single-modality approaches (Hu et al., 2021). However, this study relied on data from a single center and lacked independent external validation, leaving the generalizability of its model subject to further verification. In contrast, a study on predicting distant metastasis in non-small cell lung cancer (NSCLC) adopted a more rigorous multicenter design, constructing models based on low-dose CT radiomic and DL features, and evaluated them across internal, prospective, and external validation cohorts, demonstrating stable performance and generalizability across different datasets (Song et al., 2024). Furthermore, a novel strategy integrating quantum ML with radiomics was explored for classifying pure ground-glass nodules in small-sample datasets, showing improved precision (Huang et al., 2025). Although the concept of quantum ML is promising, the sample size was limited, and the application of quantum computing to medical image analysis remains in its nascent stages. In breast cancer, AI applications are equally robust. A diagnostic model combining DL and radiomics achieved high accuracy and strong generalizability on breast ultrasound images (Wang T. et al., 2025). A radiomics model based on multiparametric breast MRI exhibited superior performance in distinguishing benign from malignant lesions (Daimiel Naranjo et al., 2021). Another study introduced a multimodal DL framework integrating B-mode ultrasound and Nakagami parametric images, further enhancing diagnostic efficacy (Muhtadi and Gallippi, 2025). On DBT images, a radiomics-driven model enabled accurate identification of architectural distortion, potentially reducing the need for unnecessary biopsies (Chen et al., 2022).

In abdominal and pelvic malignancies, AI has also shown strong performance, though the depth of study design and validation varies considerably. A DL-enhanced radiomics model based on contrast-enhanced ultrasound effectively assists radiologists in differentiating pancreatic ductal adenocarcinoma from chronic pancreatitis (Tong et al., 2022). This study incorporated multicenter data with external validation, lending high credibility to its conclusions. In contrast, several studies on ovarian tumors (Zhou et al., 2025) or prostate cancer (Horasan and Güneş, 2024) reported high diagnostic accuracy, yet their performance metrics were derived primarily from internal test sets or cross-validation, lacking independent external validation. This distinction is critical. Models that perform well on internal test sets may overfit to the specific characteristics of the training data and often show marked performance degradation when applied to patient data from new centers, highlighting the risk of overfitting. Therefore, when evaluating model efficacy, we should prioritize studies that have undergone rigorous external validation. Even for externally validated models, clinical utility warrants further consideration. For example, a CT radiomics based ML model for automated detection of acute pancreatitis achieved diagnostic performance comparable to serum lipase levels (Bette et al., 2024), but the study did not explore how the model interacts with clinical decision making in real world emergency settings, whether it truly shortens diagnostic time, or whether it improves patient outcomes, all essential information for assessing its true clinical value. Another study successfully differentiated early-stage gastric cancer without lymph node metastasis from standard CT images using a 2.5D radiomics and DL approach (He et al., 2025).

AI-driven radiomics has also proven valuable in diagnosing diverse tumor types. For example, a clinical-radiomics-DL fusion model based on ultrasound enables accurate diagnosis of soft tissue tumors (Dai et al., 2024). The integration of radiomics and ML effectively differentiates uterine sarcomas from benign leiomyomas (Chiappa et al., 2021). A radiomics model leveraging F-18 FDG positron emission tomography (PET)/CT imaging aids in the preoperative differentiation of benign and malignant thyroid nodules (Lee et al., 2025). DL models have been employed to assist in diagnosing malignant pleural effusion (Ozcelik et al., 2023). Moreover, a dual-modality ultrasound-based hierarchical DL diagnostic model developed for unexplained cervical lymphadenopathy not only accurately identifies underlying etiologies but also reduces diagnostic variability between clinicians of differing experience levels, a benefit of particular significance in underserved and resource-limited settings (Zhu et al., 2022).

#### 4.1.2 Pathomics

Pathomics focuses on the quantitative analysis of digitized histopathological WSIs, and the application of AI in this domain provides a powerful tool for achieving automated and standardized precision diagnostics. The integration of AI with cytology and molecular biology is primarily realized through multimodal data integration and high-dimensional single-cell omics analysis. Foundational models such as scGPT enable in-depth dissection of cellular heterogeneity, developmental trajectories, and gene regulatory networks, thereby elucidating the mechanisms underlying biological processes such as cell differentiation, proliferation, and apoptosis (Heumos et al., 2023; Yiu et al., 2025). In histopathological image analysis, DL models can automatically identify cellular morphological and dynamic features, such as pseudopodia extension and light scattering changes, that are closely associated with the metastatic potential of tumor cells. By leveraging techniques such as generative adversarial networks to amplify key cellular attributes, these models enable the prediction and interpretation of complex phenotypes (Presacan et al., 2025; Zaritsky et al., 2021). To enhance model interpretability, methods such as class activation mapping (CAM) and t-distributed stochastic neighbor embedding (t-SNE) visualization are employed to uncover the biological features underlying AI decision-making, facilitating the understanding of cellular autophagy, differentiation states, and functional changes (Noorbakhsh et al., 2025; Presacan et al., 2025). Furthermore, virtual multiplex staining techniques, in combination with generative AI, enable the synthesis of multi-labeled immunohistochemical images from single H&E-stained sections. These approaches capture spatial protein expression patterns and facilitate the interpretation of intercellular interactions and functional states within the tumor microenvironment (Pati et al., 2024). AI also supports cross-scale, multi-level mechanistic modeling by integrating transcriptomic, epigenomic, proteomic, and spatial imaging data, thereby advancing precision medicine (Noorbakhsh et al., 2025; Steyaert et al., 2023; Yiu et al., 2025). Despite significant progress in AI-driven feature extraction from histopathological images, challenges remain in model interpretability, data standardization, and clinical validation (Jiang et al., 2020; Sahu et al., 2025). In CRC, a large-scale multicenter study developed a diagnostic model based on a transfer-learned deep CNN. This model demonstrated high concordance with experienced pathologists and, in certain cases, exceeded their diagnostic accuracy. It exhibited exceptional scalability in processing large volumes of slides and strong generalizability across institutions, indicating substantial potential to alleviate the diagnostic workload of pathology professionals (Wang K. S. et al., 2021). For HCC, the DL framework LiverNet enables efficient and accurate multi-class classification of HCC subtypes using H&E-stained images (Aatresh et al., 2021). Additionally, a specialized DL model facilitates precise diagnosis and quantitative assessment of microvascular invasion, thereby improving both diagnostic efficiency and inter-observer consistency (Zhang X. et al., 2024). In gastric cancer research, an innovative study revealed that histopathological features in the non-cancerous tissue surrounding the tumor possess diagnostic value. Leveraging this insight, the authors constructed a DL model capable of effectively identifying gastric cancer, highlighting the potential of contextual tissue analysis in AI-assisted diagnosis (Li L. et al., 2025). In lung cancer, the synergy between DL and pathomics holds significant promise. One study applied this approach to assess tumor invasiveness in lung adenocarcinoma (LUAD), effectively supporting junior and mid-level pathologists in improving diagnostic accuracy (Du et al., 2025). Another study developed an explainable DL-based diagnostic assistance system that not only classifies NSCLC subtypes but also generates visual explanations of its decision logic, thereby enhancing transparency and clinical trust (Civit-Masot et al., 2022). Furthermore, a pathomics-based ML model combined with clinical variables has been shown to improve the diagnostic yield of navigational bronchoscopy for peripheral pulmonary lesions (Ying et al., 2025). In hematologic malignancies, a robust framework integrating a DL-based SqueezeNet with multiple ML classifiers was proposed for the automated screening and classification of histopathological images in acute lymphoblastic leukemia, achieving exceptionally high classification accuracy (Mehan, 2025). In genitourinary cancers, a ML-based pathomics model utilizing H&E-stained images demonstrates high diagnostic accuracy for bladder cancer and generates a risk score that functions as an independent prognostic indicator (Chen S. et al., 2021). In ovarian carcinoma, a DL-based histotype diagnosis model exhibited outstanding performance and shows promise as an adjunct tool to enhance the reliability and consistency of pathological evaluations (Farahani et al., 2022).

In summary, the application of AI in radiomics and pathomics spans a broad spectrum of cancer types, from common to rare malignancies, and contributes critically to various diagnostic tasks, including lesion detection, benign-malignant differentiation, tumor grading, staging, and prognosis prediction. These technologies not only enhance diagnostic objectivity and efficiency but also hold the potential to reduce disparities in diagnostic accuracy across geographic regions and among clinicians with varying expertise, thereby contributing to more equitable and optimized allocation of healthcare resources.

### 4.2 Staging and grading

The objective and non-invasive nature of medical imaging establishes it as a cornerstone in cancer diagnosis, grading, and staging. In recent years, AI techniques, particularly traditional ML and DL, have made substantial advances in extracting quantitative features from medical images and constructing predictive models, thereby providing robust tools to support precision oncology. The following sections review the application of AI in tumor grading and staging across various imaging modalities.

#### 4.2.1 CT images

CT is widely employed for systemic tumor evaluation. CT imaging-based radiomics approaches combined with ML or DL models have demonstrated considerable potential in the grading and staging of multiple cancer types. In bladder cancer, an ML-based CT radiomics model effectively predicted muscle invasion, while a multimodal model integrating radiomic features with clinical parameters, such as age and tumor size, exhibited superior performance, significantly enhancing preoperative staging accuracy (Xiong et al., 2024). Notably, although this study achieved good performance on the test set, the sample size was relatively small (105 cases) and independent external validation was lacking, which may lead to overestimation of model performance and a risk of overfitting. Another study further revealed that the timing of CT acquisition, specifically more than 14 days post-transurethral resection, impacts model efficacy, and a model combining radiomic features with clinical biomarkers achieved an AUC of 0.82 in differentiating T2 from T3 tumors (Lisson et al., 2025). This study also had a limited sample size (97 cases) and was a single-center study, leaving its generalizability to be validated. For upper tract urothelial carcinoma, research has extended beyond the primary tumor, showing that incorporating radiomic features from both the tumor and surrounding perirenal fat substantially improves predictive performance for both tumor grade and stage, suggesting that the tumor microenvironment contains diagnostically relevant information (Al Mopti et al., 2025). However, the best performance in this study was obtained through cross-validation without validation on an independent external dataset. In CNS tumors, a study demonstrated that even conventional CT images can be leveraged by ML algorithms such as Naive Bayes to accurately differentiate low-grade from high-grade gliomas, offering a viable alternative when MRI is contraindicated or unavailable (Bilgin et al., 2025). Furthermore, in esophageal cancer, laryngeal carcinoma, and thymic epithelial tumors, fusion models that integrate CT-derived radiomic features with DL or clinical semantic data consistently outperformed single-modality approaches in grading and staging tasks (Alsallal et al., 2025; Chen et al., 2023; Zhang et al., 2025). Notably, these studies generally employed multicenter data or rigorous internal and external validation strategies. Nevertheless, the reproducibility of radiomic features remains a challenge, as variations in scanning equipment and reconstruction parameters may affect feature stability, an aspect that most studies have not thoroughly explored.

#### 4.2.2 MRI images

MRI offers superior soft tissue resolution and is the preferred imaging modality for tumor grading in the CNS, prostate, pelvis, and liver. AI models further enhance diagnostic accuracy by leveraging multiparametric MRI data. In prostate cancer, Guo et al. developed an interpretable clinical-radiomics-DL model to predict postoperative Gleason grading, with the LightGBM model achieving an accuracy of 0.848 on the internal test set (Guo et al., 2025). This study employed a two-center design and validated model performance on an external test set, strengthening the reliability of the findings. However, the sample size of the test set was relatively small (33 cases), which may lead to unstable performance estimates, and larger multicenter validation is warranted in future studies. For gliomas, multiple studies have demonstrated that based on MRI radiomics or ADC and cerebral blood flow map features, it is possible to effectively distinguish between low-grade and high-grade tumors (Chen T. et al., 2021; Du et al., 2023; Hashido et al., 2021). In meningioma grading, research has explored the diagnostic value of peritumoral edema regions and proposed a hybrid DL model integrating Vision Transformer and CNN architectures, achieving a classification accuracy of 92.86% (Zhang Z. et al., 2024). Another study confirmed that models fusing radiomic and DL features surpass the performance of unimodal systems (Yang et al., 2022). Regarding abdominal malignancies, an MRI-based radiomics model SVM outperformed a DL model DenseNet in liver tumor grading (Du et al., 2022). This study clearly partitioned training and test sets, enhancing the credibility of the results. For clear cell renal cell carcinoma (ccRCC), a ML based nomogram was constructed using multiparametric MRI data from 86 patients to predict preoperative WHO/ISUP nuclear grade, achieving an AUC of 0.933 on the test set (Yang Y. et al., 2024). Despite this strong performance, the sample size was limited and external validation was lacking. Therefore, these high AUC values should be interpreted as exploratory results requiring confirmation in larger multicenter studies. In staging applications, a T2WI MRI-based radiomics model successfully predicted lymph node burden in rectal cancer (Qubie et al., 2025), while DL models demonstrated high accuracy in automated T-staging of endometrial and tongue cancers (Lu et al., 2025; Mao et al., 2022).

#### 4.2.3 PET images

PET is usually used in combination with CT for cancer diagnosis and staging (Han et al., 2021). Amino acid PET imaging, such as 11C-Methionine (11C-MET)/CT, has shown superior capability compared to conventional MRI in delineating and grading CNS tumors. A feasibility study applied ML to radiomic analysis of 11C-MET PET/CT images, demonstrating that the resulting model could reliably differentiate between low-grade and high-grade CNS tumors across patient cohorts imaged with different scanners. This finding highlights the potential of AI-enhanced PET imaging as a non-invasive tool for tumor grading (Russo et al., 2021).

#### 4.2.4 Histopathological images

Histopathological examination remains the gold standard for cancer diagnosis and grading. AI-driven pathomics, through the analysis of digitized WSIs, aims to achieve quantitative, reproducible, and standardized tumor grading and staging. In CRC, an AI pathomics model utilizing DL-extracted features from both H&E and Ki67 immunohistochemistry slides demonstrated excellent performance in distinguishing Stage I-II from Stage III disease, with the Ki67-based model outperforming its H&E counterpart (Tan et al., 2024). This study clearly partitioned the training set (213 cases) and test set (54 cases), but lacked external validation. In renal cell carcinoma (RCC), researchers introduced RCCGNet, an efficient DL framework that enables automatic and accurate tumor grading on a novel five-tier grading dataset (Chanchal et al., 2023). Additionally, a DL model trained on WSIs showed strong agreement with expert pathologists in breast cancer grading, underscoring its potential for clinical translation (Wetstein et al., 2022).

In summary, AI, encompassing both ML and DL, has been extensively investigated for cancer grading and staging using CT, MRI, PET, and histopathological imaging. Evidence consistently indicates that multimodal models integrating radiomic or pathomic features with clinical variables achieve superior performance, highlighting the importance of data integration in advancing precision oncology.

### 4.3 Molecular classification

Molecular classification has become a cornerstone of modern oncology, providing essential insights for prognostic stratification and the selection of personalized therapeutic strategies. In recent years, AI technologies, particularly DL and ML, have demonstrated substantial potential in integrating multi-modal data to enable rapid and accurate molecular subtyping. These applications span multiple levels of biological and clinical data, including histopathological imaging, radiological features, and multi-omics profiles.

#### 4.3.1 Histopathological WSI

In the domain of pathology-based molecular classification, AI enables the direct inference of molecular characteristics from routinely stained tissue sections, thereby significantly improving diagnostic efficiency and accessibility. For diffuse gliomas, the DeepGlioma system has been developed to analyze stimulated Raman histology images within 90 s, accurately predicting key WHO-recommended molecular biomarkers, such as IDH mutation and 1p/19q co-deletion, with a mean accuracy of 93.3%, offering promise for real-time intraoperative decision support (Hollon et al., 2023). The results were obtained from a prospective, multicenter, international test cohort comprising 153 patients, lending strong generalizability and clinical relevance to the findings. In breast cancer, weakly supervised learning models can classify molecular subtypes using WSI alone and reveal subtype-specific morphological patterns, such as high-grade nuclei and tumor necrosis of triple-negative breast cancer, through attention-based heatmap (Jang et al., 2025). However, this study lacked test data from a fully independent external medical center. Similarly, for endometrial cancer (Fremond et al., 2023) and spinal cord ependymomas (Schumann et al., 2024), interpretable DL models have demonstrated high accuracy in predicting molecular subtypes based solely on H&E-stained slides, with performance occasionally exceeding that of conventional manual diagnosis.

#### 4.3.2 Radiomics

In the field of radiomics-based molecular classification, AI enables non-invasive, *in vivo* molecular characterization. Studies focusing on brain gliomas have confirmed that MRI data-based DL models can reliably predict critical molecular markers, including IDH mutation, 1p/19q co-deletion, and MGMT promoter methylation status. The application of advanced image processing techniques, such as point spread function deconvolution, can further refine radiomic features extracted from PET images, thereby enhancing the accuracy of molecular phenotyping (Ahrari et al., 2022). Moreover, “radiogenomic” models that integrate transcriptomic data with imaging features offer a novel perspective for elucidating the molecular mechanisms underlying imaging phenotypes and exhibit strong predictive performance in molecular subtyping (Shboul et al., 2021). However, the validation in this study was limited to comparative analysis on internal test sets and lacked independent testing using external data.

#### 4.3.3 Multi-omics data

At the systems level, AI harnesses clustering and ML algorithms to integrate multi-omics data, enabling the discovery of novel molecular subtypes that transcend traditional histopathological classifications and facilitating the construction of robust prognostic models. In ovarian cancer (OV), integration of multi-omics data from multi-center cohorts has led to the identification of two consensus molecular subtypes with distinct prognostic outcomes, along with the development of an ML-based signature capable of predicting immunotherapy response (Ren et al., 2025). In HCC, molecular classification combining single-cell and bulk transcriptomic data has successfully delineated patient subgroups characterized by “immune-activated” or “immunosuppressive” tumor microenvironments, providing actionable insights for tailoring immunotherapy, targeted therapy, or transarterial chemoembolization (TACE) (Chen Y. et al., 2024; Li Y. et al., 2024). Similar integrative analytical frameworks have been successfully applied to various solid tumors, including gastric cancer (Li et al., 2023; Walch et al., 2025), LUAD (Wang N. et al., 2025), and soft tissue sarcoma (Huang et al., 2022), systematically uncovering inter-subtype heterogeneity in genomic alterations, pathway activation, tumor microenvironment composition, and therapeutic sensitivity.

#### 4.3.4 Flow cytometry data

The application of AI has also expanded to the analysis of flow cytometry data (Seheult et al., 2026; Spies et al., 2025). A study on AML developed an attention-based DL pipeline capable of automated diagnosis and direct prediction of key molecular aberrations, such as PML::RARA and RUNX1:RUNX1T1 fusions and NPM1 mutations, enabling integrated analysis spanning cellular morphology to molecular classification (Lewis et al., 2024).

By extracting latent patterns from high-dimensional biomedical data, AI is fundamentally transforming the paradigms of cancer molecular classification. It not only replicates and accelerates conventional molecular testing but also uncovers previously unrecognized biological subtypes and enables precise prediction of patient prognosis and treatment response, thereby advancing the frontiers of precision oncology.

## 5 AI applications in cancer prognosis prediction

**FIGURE 4:**
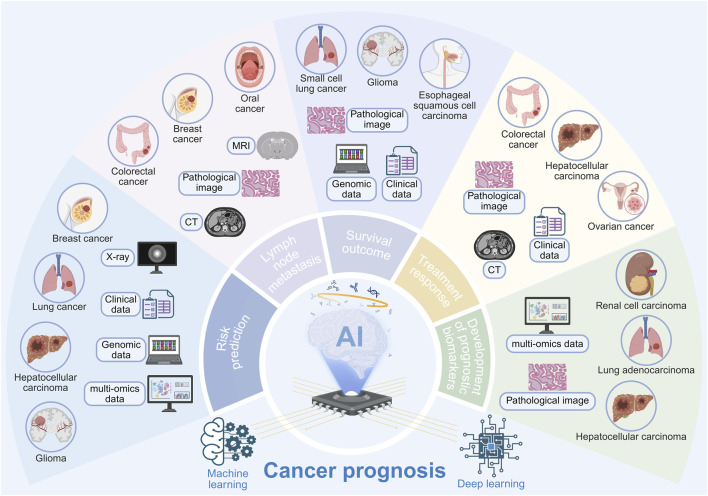
Applications of AI in cancer risk prediction, lymph node metastasis, survival outcome, treatment response and development of prognostic biomarkers. By integrating clinical data, genomic information, multi-omics indices, and imaging features, AI can effectively predict cancer risk, lymph node metastasis, and survival outcomes, while also assessing patient responses to specific therapies and aiding in the development of novel prognostic biomarkers, enabling dynamic monitoring of tumor disease progression.

Cancer prognosis assessment constitutes a cornerstone of clinical decision-making. Traditional prognostic systems rely predominantly on limited clinicopathological parameters, such as tumor stage and histological grade. However, cancer exhibits substantial inter- and intra-tumoral heterogeneity, resulting in markedly divergent treatment responses and survival outcomes even among patients classified within the same disease stage. In recent years, AI, particularly ML and DL, has opened new frontiers in deciphering tumor heterogeneity and enabling precise prognostic prediction through its advanced data integration and pattern recognition capabilities. By extracting complex, subvisual features from large-scale, multi-modal datasets, including medical imaging, histopathological WSIs, and genomic profiles, AI transforms these into quantifiable predictive indicators, thereby progressively reshaping the paradigm of cancer prognosis management (Figure 4; Table 2).

**TABLE 2:** The application of AI in cancer prognosis.

Research focus	Cancer type	AI method	Data source	Key findings	References
Risk Prediction	Breast cancer	Deep learning	Mammography	Predicted future risk based on negative screening images, demonstrating robust performance in a large UK cohort.	Ellis et al. (2024)
	Breast cancer	Deep learning	Mammography	The MAI-risk model outperformed traditional clinical models in predicting cancer risk within 5–55 months.	Romanov et al. (2023)
	Lung cancer	Machine learning	Clinical data	Achieved high prediction accuracy using MLP classifier with data augmentation techniques to address class imbalance.	M et al. (2025)
	Hepatocellular carcinoma (HCC)	Machine learning	Multi-omics data	Constructed an AI-derived risk score that served as an independent predictor for overall survival.	Wang et al. (2025d)
	Glioma	Machine learning	Genomic data	Constructed an immunogenic cell death-related risk score whose performance surpassed 71 previously published signatures.	Li et al. (2025a)
Lymph Node Metastasis	Oral squamous cell carcinoma	Deep learning	MRI	Achieved an AUC of 0.81 for predicting lymph node metastasis, significantly reduced the occult metastasis rate, with performance comparable to specialized radiologists.	Yang et al. (2025)
	Oral cancer	Deep learning	Contrast-enhanced CT	Accuracy in identifying positive lymph nodes was comparable to that of radiologists in 1466 cases.	Xu et al. (2023)
	Colorectal cancer	Deep learning	CT	A model combining deep learning and clinical features demonstrated high accuracy for preoperative prediction of lymph node metastasis.	Sun et al. (2024)
	Breast cancer	AI algorithm	Digital pathology: H&E-stained slides	Automated screening for lymph node metastasis achieved 100% sensitivity and negative predictive value for micrometastases, and isolated tumor cells.	Challa et al. (2023)
Survival Outcome	Glioma	Machine learning	Genomic data	A risk score based on a CD163+FPR3+ macrophage signature consistently predicted overall survival across six glioma cohorts.	Zhou et al. (2024)
	Small cell lung cancer	Deep learning	Histopathology images: H&E	Developed a pathomics signature that showed significant risk stratification for overall and disease-free survival.	Zhang et al. (2024b)
	Esophageal squamous cell carcinoma	Multiple ML Algorithms	Clinical features	Risk scores from models like CoxPH effectively stratified patients into groups with significantly different 3-year overall survival rates.	Zhang et al. (2023b)
Treatment Response	Colorectal cancer	Multistain deep learning	Histopathology images	The AImmunoscore predicted response to neoadjuvant therapy in rectal cancer patients.	Foersch et al. (2023)
	Colorectal cancer	Deep learning	Histopathology images: H&E	A pathomics signature identified stage II/III patients more likely to benefit from adjuvant chemotherapy.	Lou et al. (2025)
	HCC	Machine learning	Clinical data	The CLAM-C model predicted differential responses to various treatments in BCLC-C stage patients.	Han et al. (2024)
	HCC	Deep learning	CT	A model predicted survival after TACE in intermediate-stage HCC patients; those with lower scores benefited more.	Wang et al. (2022)
	Ovarian cancer	Graph deep learning	Histopathology images: H&E	The OCDPI model predicted response to adjuvant therapy; patients with low scores had better survival and lower recurrence.	Yang et al. (2024b)
Prognostic Biomarker Development	Urothelial carcinoma	Machine learning	Multi-omics data	Identified a consensus machine learning signature of 12 genes that robustly predicted prognosis and immunotherapy response.	Chu et al. (2023)
	Renal cell carcinoma	Machine learning	Multi-omics data	Constructed a prognosis and treatment vulnerability signature to guide personalized treatment strategies.	Chi et al. (2025)
	Lung adenocarcinoma	Deep learning	Histopathology images: whole slide	A deep learning-defined risk group was an independent predictor of disease-free survival, complementing TNM staging.	Chen et al. (2025c)
	HCC	Machine learning	Multi-omics data	Constructed a cell death-related index based on programmed cell death patterns, predicting prognosis and treatment response.	Yan et al. (2025b)

### 5.1 Risk prediction

AI models, particularly those based on DL, have been widely applied to predict an individual’s risk of developing cancer using medical images and clinical data. In breast cancer risk prediction, DL models trained on screening mammography images demonstrate strong predictive performance. One study developed an AI model capable of predicting future breast cancer risk based solely on currently negative mammograms. This model exhibited robust performance in a large, representative UK screening cohort, with consistent performance across different sites, ethnicities, and age groups, underscoring its potential for broad population-level implementation (Ellis et al., 2024). Another study employed a DL architecture enhanced with an attention mechanism for risk prediction from mammographic images, outperforming conventional clinical risk models in forecasting cancer development within 5–55 months (Romanov et al., 2023). For lung cancer, ML-based risk prediction models have been constructed by applying data augmentation techniques to mitigate class imbalance in clinical datasets, combined with optimized classifiers, achieving high accuracy and offering a valuable tool for early screening (M et al., 2025). In HCC, the integration of multi-omics data with ML algorithms enables the identification of molecular subtypes with distinct prognoses and facilitates the construction of an AI-derived risk score. This score functions as an independent predictor of overall survival and holds promise for guiding personalized therapeutic strategies (Wang Z. et al., 2025). In glioma, a study constructed a risk score based on immunogenic cell death-related genes, which effectively predicted patient prognosis and demonstrated superior performance compared to previously published signatures, establishing itself as a promising novel biomarker (Li G. et al., 2025).

### 5.2 Lymph node metastasis

AI demonstrates significant potential for non-invasive, preoperative prediction of lymph node metastasis, supporting clinical decision-making and surgical planning by reducing both unnecessary lymph node dissections and missed metastatic lesions. In oral squamous cell carcinoma, a three-stage DL model based on MRI was developed to detect lymph node metastasis. The model achieved high accuracy in external validation cohorts and significantly reduced the rate of occult metastases. Its diagnostic performance was comparable to, and in some cases superior to, that of specialized radiologists, providing robust evidence for precision surgical planning (Yang et al., 2025). Another study on oral cancer utilized contrast-enhanced CT and transfer learning to develop a DL model. In a cohort of 1,466 cases, the model achieved accuracy in identifying positive lymph nodes comparable to that of radiologists and substantially higher than that of surgeons and medical students, demonstrating its reliability as an auxiliary diagnostic tool (Xu et al., 2023). However, the validation set was likely derived from the same institution, raising the potential risk of overfitting. In CRC, a CT-based DL radiomics model was developed for preoperative prediction of lymph node involvement. By integrating DL-extracted features with a clinical model, it achieved high predictive accuracy and demonstrated effective risk stratification for predicting progression-free survival (Sun et al., 2024). In breast cancer, an AI algorithm was integrated into the clinical digital pathology workflow to automatically screen H&E-stained sections for lymph node metastasis. The algorithm achieved perfect sensitivity and negative predictive value, serving as an efficient screening tool that substantially enhances pathologists’ workflow efficiency (Challa et al., 2023).

### 5.3 Survival outcomes

AI models can extract discriminative features from histopathological images, radiomic profiles, and multi-omics data to accurately stratify patients according to survival outcomes, often surpassing the predictive power of traditional staging systems. A study leveraged ML algorithms to develop a prognostic model based on a macrophage-related gene signature, whose risk score consistently predicted overall survival across multiple independent glioma cohorts, outperforming conventional clinical variables (Zhou et al., 2024). This cross-cohort consistency validation substantially reduced the risk of overfitting and enhanced the robustness of the model. In small cell lung cancer (SCLC), research utilizing H&E-stained histopathological images identified distinct histomorphological phenotype clusters, which were integrated into a pathomics signature with significant prognostic value. This signature enabled robust risk stratification for both overall and disease-free survival in independent multicenter cohorts, providing prognostic information beyond TNM staging and existing molecular classifications (Zhang Y. et al., 2024). In ESCC, a comparative analysis of 6 ML algorithms for survival prediction revealed that risk scores derived from top-performing models effectively stratified patients into groups with significantly different 3-year overall survival rates, with predictive accuracy exceeding that of traditional staging systems (Zhang K. et al., 2023).

### 5.4 Treatment response

Predicting individual patient response to specific therapies is critical for delivering personalized care and avoiding ineffective treatments and adverse effects. AI has demonstrated considerable utility in this domain. In CRC, a study established a multistain DL model to compute an immunoscore, which not only predicted prognosis but also showed potential in forecasting rectal cancer patients’ response to neoadjuvant therapy (Foersch et al., 2023). Another study developed a DL-based pathomics signature from H&E images and found that patients with low signature scores were more likely to benefit from chemotherapy, offering actionable insights for adjuvant treatment decisions (Lou et al., 2025). In the context of HCC, a recent study employed ML to develop a risk stratification model (CLAM-C) for patients diagnosed with advanced-stage HCC according to the Barcelona Clinic Liver Cancer stage C (BCLC-C). The CLAM-C model demonstrates the capacity to predict differential responses to key therapeutic modalities, including immune checkpoint inhibitors, transarterial therapies, and tyrosine kinase inhibitors, thereby supporting more precise treatment selection in clinically complex scenarios (Han et al., 2024). Another study developed a CT-based DL model to predict survival outcomes in intermediate-stage HCC patients following TACE. Patients with lower model scores derived greater survival benefit from TACE, suggesting its utility as a practical tool for patient selection (Wang et al., 2022). In OV, a graph-based DL model was developed using H&E WSI to predict both prognosis and response to adjuvant therapy. Patients with low model scores exhibited improved survival outcomes and lower recurrence rates after adjuvant treatment, indicating the model’s potential to identify therapy-sensitive subpopulations (Yang Z. et al., 2024).

### 5.5 Developing prognostic biomarkers

By analyzing high-dimensional omics data, AI enables the discovery and validation of novel, interpretable prognostic biomarkers. These digital biomarkers are emerging as key components of precision oncology. Multiple studies integrate multi-omics data with various ML algorithms to derive robust, consensus-driven ML signatures or risk scores that serve as powerful prognostic indicators. For example, in muscle-invasive urothelial carcinoma, multi-omics clustering combined with ML identified a gene signature that reliably predicted both prognosis and response to immunotherapy (Chu et al., 2023). In ccRCC, integrative analysis of multi-center multi-omics data yielded a composite signature predictive of prognosis and therapeutic vulnerability, with potential to guide individualized treatment planning (Chi et al., 2025). AI models applied to digital pathology images can extract deep morphological features from routine H&E slides and convert them into quantifiable biomarkers. In LUAD, for instance, the risk group defined by a WSI-based DL model served as an independent predictor of disease-free survival, complementing conventional TNM staging (Chen T. et al., 2025). Furthermore, AI can transform biological processes into measurable biomarkers. One study classified HCC based on patterns of PCD and used ML to construct a cell death-related index, which predicted patient prognosis and treatment response, highlighting the role of cell death pathways in tumor heterogeneity (Yan X. et al., 2025).

In summary, AI, by deeply mining the rich information embedded in medical imaging, digital pathology, and multi-omics data, has achieved transformative advances in multiple dimensions of cancer prognosis, including risk prediction, lymph node metastasis assessment, survival stratification, treatment response forecasting, and the development of novel digital biomarkers. These innovations not only enhance the accuracy and personalization of prognostic evaluation but also hold the potential to fundamentally reshape clinical decision-making, driving oncology toward a more precise, efficient, and patient-centered future.

## 6 Challenges and prospects

Although AI has demonstrated substantial potential in cancer diagnosis and prognosis prediction, its clinical translation still faces multiple challenges (Bi et al., 2019; Huang et al., 2020).

At the data level, quality and standardization represent the primary bottleneck. Current medical data are characterized by widespread multicenter heterogeneity and inconsistent annotation standards, which severely limit model generalizability (Willemink et al., 2020; Zech et al., 2018). The clinical setback of IBM Watson for Oncology serves as a cautionary reminder that the diversity, breadth, and real-world representativeness of training data are fundamental to model robustness (Pennestrì et al., 2025). Therefore, there is an urgent need to establish unified data standards, promote high-quality data sharing, and ensure population diversity during model development.

At the model level, technological limitations require urgent attention. First, multimodal data fusion remains a research challenge. Heterogeneous data such as MRI, CT, and histopathological images differ substantially in resolution, dimensionality, and information content. Designing optimal fusion strategies to synergistically integrate information while avoiding redundancy and noise interference warrants further investigation (Acosta et al., 2022; Lipkova et al., 2022). Second, although DL achieves remarkable performance, its “black box” nature and poor interpretability constrain clinical trust. While some studies have attempted to open the black box using techniques such as attention heatmaps, these methods often provide correlational rather than causal explanations and must be interpreted with caution (Ghassemi et al., 2021). Furthermore, models need to be validated across diverse populations to assess performance differences across races, geographic regions, and healthcare resource settings. Studies have shown that AI models trained predominantly on European or American populations exhibit significant performance degradation, such as increased false-positive rates, when applied across racial groups (Nguyen et al., 2024).

At the clinical practice level, human-AI collaboration and doctor-patient communication present new challenges. AI systems must be seamlessly integrated with electronic medical records and imaging systems to deliver real-time, precise, and easily interpretable decision support, rather than merely providing another binary classification output (Kelly et al., 2019). Moreover, explaining the role and limitations of AI to patients in accessible language, thereby avoiding misunderstanding or unrealistic expectations, represents an ethical and communication imperative for future healthcare (Richardson et al., 2021). In parallel, appropriate ethical and regulatory frameworks must be established to ensure fair, equitable, and responsible application of AI technologies (Char et al., 2018; Wu et al., 2021).

This review primarily focuses on the applications of AI in cancer screening, diagnosis, and prognosis prediction. However, we recognize that AI also demonstrates significant potential in cancer treatment optimization, such as radiotherapy planning (Giraud and Bibault, 2024; Vandewinckele et al., 2020) and prediction of immunotherapy response (Kong et al., 2022; Prelaj et al., 2024). In addition, AI is increasingly being applied in cancer drug discovery, encompassing areas such as target identification (Bi et al., 2025; Noor et al., 2023), compound screening (Fu and Chen, 2025; Manan et al., 2025), and drug repurposing (Issa et al., 2021), thereby enhancing the efficiency of novel drug development. Moreover, AI tools are being increasingly integrated into multidisciplinary tumor boards (MTBs), supporting decision-making, data integration, and workflow optimization (Erdat et al., 2025; Vela Ulloa et al., 2023; Zabaleta et al., 2025), while also showing preliminary applications in palliative care (Bozkurt et al., 2025; Gajra et al., 2022), psychological support (Li K. et al., 2025), and doctor-patient communication (Chen D. et al., 2025; Wu et al., 2026) for patients with terminal cancer. To overcome the limitations of data dependency and privacy constraints, emerging training paradigms such as self-supervised learning, contrastive learning, and federated learning are gaining traction. These approaches enable models to learn robust representations from unannotated data across institutions without compromising patient privacy (Krishnan et al., 2022; Rieke et al., 2020). Although these directions are still in the exploratory stage, their development is expected to further highlight the value of AI in comprehensive cancer management.

## 7 Conclusion

AI technology is profoundly transforming the paradigms of cancer diagnosis and prognostic prediction. From ML- and DL-based medical image analysis to the processing of clinical text using LLMs, AI has demonstrated considerable potential and practical value across various cancer types in screening, diagnosis, molecular classification, grading, staging, and outcome prediction. Evidence suggests that AI models can not only achieve performance comparable to that of human experts but in certain cases exceed it, while also identifying subtle patterns in routine clinical data that are undetectable to human observers, enabling capabilities such as “digital biopsy” and refined risk stratification. Moreover, multimodal AI models that integrate diverse data sources, including imaging, omics, and clinical records, typically exhibit superior predictive performance. The integration of AI into oncology holds significant promise for improving diagnostic objectivity, efficiency, and accuracy, as well as optimizing the allocation of healthcare resources.
